# The impact of the *Prime Time Sister Circles*® (PTSC) on blood pressure of low-income mid-life African American women in the United States

**DOI:** 10.1057/s41271-023-00450-5

**Published:** 2023-10-29

**Authors:** Darrell J. Gaskin, Hossein Zare, Chidinma A. Ibe, Manshu Yang, Wehmah Jones, Marilyn Gaston, Gayle Porter, Denise L. Woods, Michele Balamani, Nicole Jones, Vivienne A. Rose, Richard Allen Williams, Charles Rohde

**Affiliations:** 1 Johns Hopkins Center for Health Disparities Solutions, Johns Hopkins University, Baltimore, MD USA; 2 Department of Health Policy and Management, Johns Hopkins Bloomberg School of Public Health, Baltimore, MD USA; 3 Department of Global Health Services and Administration, University of Maryland Global Campus, Adelphi, MD USA; 4 Division of General Internal Medicine, Department of Medicine, Johns Hopkins University School of Medicine, Baltimore, MD USA; 5 Department of Psychology, University of Rhode Island, Kingston, RI USA; 6 American Institutes for Research, Washington, DC USA; 7 The Gaston & Porter Health Improvement Center, Inc., Washington, DC USA; 8 Baraka and Associates, Largo, MD USA; 9 Baltimore Medical System Inc., Baltimore, MD USA; 10 David Geffen School of Medicine, University of California Los Angeles (UCLA), Los Angeles, CA USA; 11 Department of Biostatistics, Johns Hopkins Bloomberg School of Public Health, Baltimore, MD USA; 12 Department of Health Policy and Management, Johns Hopkins University Bloomberg School of Public Health, 624 North Broadway Ste 441, Baltimore, MD 21205-1900 USA

**Keywords:** Community-based behavioral intervention, Hypertension, BMI, African American women

## Abstract

There is a pressing need to develop and evaluate culturally tailored, community-based interventions that address hypertension management among low-income African American women. We employed a randomized controlled trial to test the effectiveness of the *Prime Time Sister Circles® Program* in reducing blood pressure and body mass index among low-income African American women ages with hypertension. Study participants (*N* = 339) were African American women aged 40–75 years who were diagnosed with hypertension and received their primary care at government funded health centers in Washington, D.C. Compared to the usual care group, *Prime Time Sister Circles®* participation was associated with a reduction in systolic BP by − 2.45 (CI − 6.13, 1.23) mmHg, a reduction in diastolic BP by − 3.66 mmHg (CI − 6.32, − 0.99), and a change in BMI by − 0.26 (CI − 2.00, 1.48) from baseline to 15 months. The results suggest that culturally tailored community-based interventions can improve hypertension management in low-income women.

**Supplementary Information:**

The online version contains supplementary material available at 10.1057/s41271-023-00450-5.

## Key messages

- The *Prime Time Sister Circles*®* Program*, a culturally tailored community-based intervention, can improve blood pressure management for low-income African American women with hypertension.
- Lifestyle changes that improve health behaviors among low-income women can be achieved with culturally tailored community-based interventions.

## Introduction

African American women experience higher rates of hypertension and obesity compared to women from other racial and ethnic groups [1]. According to the United States (US) National Center for Health Statistics (NCHS) in 2013–2016, 42.9% of African American women aged 20 and over had hypertension. Among these women, 57.7% had uncontrolled blood pressure [2]. Similarly, in 2017–2018, the NCHS reported that 56.9% of African American women aged 20 and over were obese. Hypertension and obesity are risk factors for heart disease, stroke, diabetes, vascular dementia, and chronic kidney disease, which are the leading causes of death for African American women. Fortunately, hypertension and obesity can be managed with lifestyle changes (such as diet, exercise, improved stress management) and medication [3]. Systematic reviews of four randomized trials, one quasi-experimental design, and four pre-post studies of community-based cardiovascular health interventions in marginalized populations show mostly positive results for African Americans [4]. Community-based interventions can help women reduce their blood pressure and lose weight; there is a need, however, for culturally tailored lifestyle modification programs that meet the unique needs and concerns of African American women [5]. This is critical considering evidence placing intersecting social risk factors, including discrimination, stress, and poverty, in the causal pathways of hypertension incidence and suboptimal management among African American women [6–8].

Studies of a few culturally tailored community-based interventions have shown them to be effective at addressing risk factors for cardiovascular disease among well-educated, mid-life African American women. These interventions typically provide health information about chronic health conditions and focus on improving diet, physical activity, and medication adherence. They combine a health education curriculum prepared for the general public with culturally sensitive setting, delivery, and reinforcements to appeal to mid-life and older African American women. Four studies provide evidence that community-based, culturally tailored interventions can improve blood pressure control and reduce weight among mid-life and older African American women [9–12]. However, two studies report that culturally tailored inventions were not more effective than usual care [13, 14].

There is a dearth of interventions focused exclusively on mid-life African American women [15]. However, a pre–post study of the *Prime Time Sister Circles®* (*PTSC*) *program*, a promising community-based intervention demonstrated some efficacy at helping African American women control their blood pressure and weight in community-based settings. In a sample of 656 African American women from Washington, D.C., Tampa, Florida, and Baltimore, Maryland, Thomas, and colleagues observed that among *PTSC* participants, the proportion of those with blood pressure in the hypertensive range (> 140/90) declined from 39.1% at baseline to 28.8% at 12 weeks. This improvement was sustained in 6 months. They also reported that participants in the PTSC program exhibited lower levels of stress, increased engagement in physical activity, and improved eating patterns [16]. The *PTSC program* has received numerous awards for its innovative approach to health behavior change among African American women, including the Encore’s Purpose Prize Winners for Social Innovation; the Johnson and Johnson’s Women’s Health Leadership Award; the “Healthy Heart Award” from the US National Institutes of Health (NIH) and the American Heart Association (AHA), in conjunction with the “Red Dress Day Award” from Woman’s Day Magazine; and the International Black Women’s Congress Lifelong Commitment to Black Women’s Health Award.

This study presents the results of a randomized clinical trial featuring the *PTSC* program (PTSC-RCT) that prioritized less-educated and lower income African American women. We assess whether this program improved the blood pressure (BP), BMI, and health behaviors (that is, healthy diet, increased physical activity, and stress management) of low-income mid-life African American women with hypertension whose primary source of care was at a federally qualified health center (FQHC). A FQHC is a community health center that receives government funds to provide services to low-income patients in underserved communities.

## Methods

### Trial design

This study analyzes data collected as part of a larger randomized clinical trial that evaluates the effectiveness of the *Prime Time Sister Circles®* (PTSC) on health, health care utilization, and behavioral outcomes. Details of the PTSC-RCT have been provided elsewhere [17, 18]. The PTSC-RCT’s primary outcome was hypertension control, and it aimed to assess the impact of the PTSC intervention on hypertension. The study’s secondary outcomes include BMI, health knowledge, self-efficacy, behaviors, and psychosocial health. We originally specified the health behaviors (dietary and physical activities) as secondary outcomes, but the nature of the measures makes them more appropriate as process measures.

### Participants

Data for this study came from the PTSC-RCT. As a community-based intervention, the PTSC recruited English-speaking women who self-identified as African American, between 40 and 75 years of age, with a history of uncontrolled hypertension diagnosis (systolic blood pressure ≥ 140 mm Hg and diastolic blood pressure ≥ 90). We identified women as uncontrolled if diagnosed with hypertension and deemed resistant to treatment by their health provider, as evidenced by elevated BP (> 140/90) during their last visit. We selected women who received their care from a FQHC, Unity Health Care, and who resided in Wards 6, 7, and 8, the highest poverty Wards in Washington, D.C. Women received an invitation letter from the Medical Director describing the study, incentives for participation, contact information for pursuing enrollment, and the dates and locations for recruitment meeting sessions to learn about the PTSC program and the study at large. We collected baseline data from those who subsequently agreed to participate and a signed, written consent. We randomly assigned participants to the intervention (PTSC group) or control group (usual care group). We collected data from July 2017 through June 2020.

### Interventions

The PTSC is a multifaceted, theory-driven, and community-based intervention characterized by didactic training delivered by trained African American facilitators, content experts (for hypertension, stress management, fitness, and nutrition), and intensive peer-based social support. Marilyn Gaston (a physician) and Gayle Porter (a clinical psychologist) established The Gaston and Porter Health Improvement Center, Inc. (GPHIC), as a National Training Institute. The GPHIC trained PTSC facilitators on cardiovascular conditions, group facilitation and leadership, and on cultural beliefs undergirding self-management of chronic conditions. The facilitators were PTSC program graduates who resided in the communities the program serves [17]. Each Sister Circle is composed of 25–30 mid-life African American women (with mid-life defined as ages 40–75, in accordance with Gaston and Porter’s designation). The intervention targets this age group to as an opportunity for these women to make “mid-course health corrections” in the hopes of avoiding cardiovascular disease complications emerging from hypertension.

PTSC addresses three key modifiable health risk factors for chronic disease: unmanaged stress, physical inactivity, and unhealthy nutritional choices. It addresses additional risk factors that contribute to unhealthy lifestyles: lack of knowledge or misinformation about major illnesses, including hypertension; and it also aims to encourage mid-life African American women to prioritize their health and take proactive steps of primary and secondary prevention to manage their health and health care outcomes. PTSC is structured to support African American women to deepen their grasp of the requisite information, motivation, skills, and consultative support to improve and maintain their health. They are assisted in developing habits of primary and secondary prevention.

Those assigned to the PTSC intervention group attended a two-hour per week facilitator-led Sister Circle for 13 weeks. Sessions featured an educational presentation provided by facilitators or experts on the topic of the week. Facilitators discussed the topics in relation to hypertension and cardiovascular disease and included overall disease prevention, stress management, mental health (depression and anxiety), self-esteem, fitness, nutrition, psychosocial well-being, and effective communication with health care team members. Following the presentations, facilitators guided discussion with all participants, including role playing to model how best to navigate circumstances that could threaten their ability to adopt the recommended lifestyle change (for example, talking with their family members about the changes they are making because of the PTSC, or discussing with their physician and asking health-related questions).

### Outcome variables

The study’s primary outcomes included systolic and diastolic blood pressure. The secondary outcomes were BMI (defined as a person’s weight in kilograms divided by the square of height in meters) and stress management techniques. To explain the changes in BP and BMI, we collected self-reported physical activity, and dietary behaviors as process measures. Trained research team members measured study participants’ BP, height, and weight and administered surveys at baseline, 3 months, 9 months, and 15 months. These research team members were typically graduate students in the school of public health with medical or nurse training. The self-reported survey covered many other items, including but not limited to self-rated health, health care utilization, and health behaviors. We designed the survey to be self-administered, but we provided additional support to participants who had difficulty reading. A trained assistant read the survey item by item and recorded the participant’s response. We previously described some of these outcomes [17].

### Covariates for physical activity, eating habits, stress, and stress management

To measure physical activity, we used participants’ responses to whether they exercised for 30 min per day or 150 min per week, did strength training at least twice a week, and used a pedometer in the past month. This was a process measure to explain changes in the primary (systolic and diastolic BP) and secondary outcomes (BMI).

We assessed eating habits through a self-report. Participants indicated whether, in a typical day, they ate five servings of vegetables, ate four servings of fruit, watched their portion size, and read the food labels. We also captured their emotional state upon eating (that is, if they ate when full, upset, or not hungry); the type of food consumed (fast foods, snacks, fried foods, or sweets); and beverage consumption (such as non-diet soda). We measured each of these items at baseline, 3 months, 9 months, and 15 months. This is also a process measure.

We measured stress through the Perceived Stress Scale (PSS) [19]. We measured stress management through items focused on techniques they employed to reduce stress. To capture dimensions of stress management strategies, we created two scales: an 11-item scale appraising adaptive techniques (listen to music, pray, exercise, deep breathing, talk with family or friends, meditate, spend time with friends, yoga, talk with minister, talk with therapist, and massage) and a 7-item scale encapsulating non-adaptive techniques (shop, watch television, eat, drink alcohol, smoke cigarettes, avoid it, complain about it). We computed the adaptive technique score by summing the number of techniques used, ranging from 0 to 11. And for the non-adaptive techniques score, we summed the number of techniques not used, ranging from 0 to 7.

### Control variables

We adjusted our estimates for participants’ sociodemographic information: age, marital status (single without partner, married or living with a partner, other status, divorced, separated or widowed), level of educational attainment (less than high school, high school graduate, more than high school), income (under $20,000, $20,001–$40,000, and above $40,000), having any kind of health insurance, currently smoking, currently drinking, health literacy, and numeracy [20, 21].

### Sample size

We excluded 2 participants with missing responses on blood pressure, which reduced the analytical sample to 339. Of the 339 participants, 215 were randomly assigned to receive the PTSC intervention, and 124 were assigned to the usual care group. Of note, 54 participants (25%) assigned to the PTSC intervention did not participate primarily because it was offered at a time or location that was inconvenient for them, although we were able to collect their follow-up data. We designated these participants, who did not receive the intervention as intended, as the Intent-to-Treat (ITT) group (see CONSORT diagram for more details) and then referred to them as the “ITT sample.” To offset the impact of suboptimal intervention receipt among those who make up the ITT sample, we randomly assigned more women to the intervention group (at a 2:1 ratio) to ensure adequate numbers of women completing the PTSC intervention (Supplementary Material Fig. S1).

## Statistical analysis

We collected data at baseline, 3 months, and 15 months. We compared the outcomes and population characteristics of the PTSC group (intervention), the intent-to-treat (ITT) group, and usual care group (control) group, using mean and standard deviation (SD) for continuous data, and counts and percentages for categorical data. We conducted unequal variances *t* tests to detect statistically significant differences between the treatment arms. To assess the impact of the PTSC program on participants’ blood pressure (systolic and diastolic) and BMI, we used balanced panels between baseline and 3 months, baseline and 9 months, and baseline and 15 months. We employed a difference-in-differences methodology to estimate the effectiveness of PTSC on blood pressure, BMI, and health behaviors. We conducted both intention-to-treat and per protocol analyses [15, 22, 23]. Due to the presence of missing data for education, income, marital status, health insurance coverage, drinking and smoking status, we used multiple imputation techniques, a simulation-based statistical techniques to handle data missing assuming data are missing at random. To account for the potential bias resulting from the ITT group, who were assigned to the intervention but did not attend their Sister Circle sessions, we included them in the analysis as part of the PTSC group in the intention-to-treat analysis but excluded them in the per protocol analysis (see Supplementary Material Tables S1–S4). We conducted all statistical procedures using Stata, version 15. The Institutional Review Boards (IRBs) of the Johns Hopkins Bloomberg School of Public Health (JHBSPH) and the American Institutes for Research approved the PTSC-RCT (JHBSPH IRB No. 00007121).

## Results

Table 1 displays the clinical outcomes, demographic characteristics, socioeconomic factors, health behaviors, health literacy, and numeracy at baseline for the PTSC, ITT and usual care samples. At baseline and for all participants, the mean SBP was 132.8 and DBP was 84.5. Their mean BMI was 34.8, indicating that they were, on average, in the obese category. The average age was 57.0. Most participants were single without a partner (56%). Almost half reported currently drinking alcohol or smoking cigarettes. The women had low health literacy, scoring an average of 4.3 relative to a maximum of 12; and moderate numeracy, scoring 11.2 relative to a maximum of 18). This is a low socioeconomic sample with about 30% of women without a high school education, and 40% who did have a high school diploma/graduate equivalency diploma (GED). Over 71% of the participants’ annual household income fell below $20,000. Most (78%) participants were covered by Medicaid, a public insurance program for poor and persons living with disabilities in the US.

**Table 1:** Distribution of PTSC-RCT study population

Clinical outcomes	Total		Usual care		PTSC		ITT	
	*N*	Mean (SD) or %	*n*	Mean (SD) or %	*n*	Mean (SD) or %	*n*	Mean (SD) or %
Systolic blood pressure (SBP)	339	132.81 (20.3)	124	133.26 (20.27)	161	131.23 (19.07)	54	136.50 (23.56)
Diastolic blood pressure (DBP)	339	84.55 (12.51)	124	86.08 (12.42)	161	83.77 (12.46)	54	83.35 (12.78)
Body mass index (BMI)	339	34.83 (9.09)	124	35.76 (8.84)	161	34.88 (9.15)	54	32.53 (9.24)^a^
Age (years)	339	57.03 (8.14)	125	56.61 (8.33)	162	57.14 (7.68)	52	57.71 (9.13)
Marital status (%)								
Single without a partner	191	56.01	72	57.60	95	58.64	24	44.44
Married or live with a partner	67	19.65	22	17.60	31	19.14	14	25.93
Separated, divorced, or widowed	74	21.70	29	23.20	33	20.37	12	22.22
Missing	9	2.64	2	1.60	3	1.85	4	7.41
Education (%)								
Less than high school	83	24.34	27	21.60	47	29.01	9	16.67
High school graduate/GED	137	40.18	56	44.80	60	37.04	21	38.89
More than high school	93	27.27	33	26.40	43	26.54	17	31.48
Missing	28	8.21	9	7.20	12	7.41	7	12.96
Income (%)								
Under $20,000	242	70.97	91	72.80	122	75.31^b^	29	53.70^a^
$20,001–$40,000	60	17.60	23	18.40	30	18.52	7	12.96
$40,001–above	15	4.40	6	4.80	6	3.70^b^	3	5.56
Missing	24	7.04	5	4.00	4	2.47	15	27.78^a^
Has health insurance (%)	315	96.04	120	97.56	150	96.15	45	91.84
Private health insurance	2	0.59	0	0.00	1	0.62	1	1.85
Medicaid	265	77.71	103	82.40	127	78.40	35	64.81
Missing	7	2.05	1	0.80	3	1.85	3	5.56^a^
Currently smoke (%)	144	43.37	53	43.80	64	40.00	27	52.94
Missing	9	2.64	4	3.20	2	1.23	3	5.56
Drink alcohol (%)	154	49.20	51	44.35	74	48.05^b^	29	65.91^a^
Missing	28	8.21	10	8.00	8	4.94^b^	10	18.52
Health literacy and numeracy								
Health literacy scale	331	4.34 (2.26)	120	4.32 (2.11)	159	4.30 (2.28)	52	4.50 (2.51)
Numeracy scale	338	11.17 (4.52)	124	11.53 (4.54)	162	10.84 (4.33)	52	11.31 (5.00)

The values are represented with ± means ± SD. All binary variables are coded 1 and 0 represents the name of the variable. There is no significant difference between the usual care (UC) and PTSC group

^a^UC vs. intent-to-treat < 0.05

^b^PTSC vs. intent-to-treat < 0.05

The randomization worked reasonably well with 161 participants in the PTSC group, 54 participants in the ITT group, and 124 participants in the usual care group. There were few statistically significant differences between the three groups at baseline. There were no differences in the clinical outcomes, except for lower BMI in the ITT group. There were no demographic and socioeconomic differences, except for higher percentages of participants in the PTSC group with a high school diploma/GED and enrolled in Medicaid. Higher percentages of the ITT group reported smoking and drinking, but the baseline health behaviors of the usual care and PTSC groups were similar. There were no differences in health literacy and numeracy.

We surveyed the women who participated in the PTSC group to assess their levels of satisfaction with the intervention. About 92% of participants reported satisfaction with the PTSC program. Over 90% of the participants agreed that the facilitators, content experts, and curriculum materials were effective. They reported that the Sister Circle conferred additional knowledge and skills to help them achieve their health-related goals and that participation in the Circles encouraged them to prioritize their health.

We display results from the fixed effects models in Table 2 suggesting a negative association between PTSC participation and SBP and DBP at 3 months, 9 months, and 15 months. Although, these associations were not always statistically significant, the direction was consistent across the study period. Compared to the usual care group, PTSC participation was associated with reductions in systolic BP by − 2.35 (CI − 6.04, 1.33) mmHg and diastolic BP by − 1.94 (CI − 4.32, 0.43) from baseline to 3 months, reductions in systolic BP by − 1.19 (CI − 4.84, 2.46) mmHg and diastolic BP by − 0.96 (CI − 3.23, 1.31) from baseline to 9 months; and reductions in systolic BP by − 2.45 (CI − 6.13, 0.94) mmHg and diastolic BP by − 3.66 (CI − 6.32, − 0.99) from baseline to 15 months. We found no association between PTSC participation and change in BMI at any of the three-time intervals. The results were similar for the per protocol analysis. There is a statistically significant change in diastolic BP by − 3.66 (CI − 6.32, − 0.99) from baseline to 15 months.

**Table 2:** Impact of PTSC participation on change in systolic and diastolic blood pressure at 3 months, 9 months, and 15 months for hypertensive mid-life African American women who use an FQHC for their primary care

Intention-to-treat analysis	SBP	DBP	BMI
	*b*/SE	*b*/SE	*b*/SE
Baseline vs. 3 months			
PTSC and ITT	− 2.35	− 1.94	− 0.47
	[− 6.04 to 1.33]	[− 4.32 to 0.43]	[− 2.14 to 1.21]
*N*	226	226	227
Baseline vs. 9 months			
PTSC and ITT	− 1.19	− 0.96	− 0.71
	[− 4.84 to 2.46]	[− 3.23 to 1.31]	[− 2.35 to 0.94]
*N*	231	231	231
Baseline vs. 15 months			
PTSC and ITT	− 2.45	− 3.66*	− 0.26
	[− 6.13 to 1.23]	[− 6.32 to − 0.99]	[− 2.00 to 1.48]
*N*	188	188	188

The usual care group is the reference category. We controlled for age, marital status, educational attainment, income, health insurance coverage, smoking and drinking behavior, health literacy, and numeracy. We have used multiple imputation methods to fill in missing observations for all covariates

*SBP* systolic blood pressure, *DBP* diastolic blood pressure, *BMI* body mass index

*p < 0.01

There is some evidence that PTSC participation increased physical activity at 3 months and 9 months, but that it disappeared at 15 months. Compared to the usual care group, PTSC participants were more likely to increase strength training and pedometer use by 0.09 (CI 0.00, 0.19) and 0.10 (CI 0.01, 0.11) at 3 months, respectively. The effect on strength training was sustained at 9 months from baseline, 0.12 (CI 0.02–0.21). By 15 months, the effect on both physical activity measures had dissipated. There was no effect on exercising for at least 30 min (Table 3.) PTSC participants were more likely to watch their portion size and read food labels at 3 months than their counterparts in the usual care group, but these differences disappeared by 15 months. Participants did not change their consumption of vegetables and fruit relative to the usual care group (Table 4).

**Table 3:** Impact of PTSC participation on change in physical activity at 3 months, 9 months, and 15 months for hypertensive mid-life African American women who use an FQHC for their primary care

Intention-to-treat analysis	Exercise	Strength training	Pedometer use
	*b*/SE	*b*/SE	*b*/SE
Baseline vs. 3 months			
PTSC and ITT	− 0.03	0.09	0.10*
	[− 0.12 to 0.05]	[− 0.00 to 0.19]	[0.01 to 0.19]
*N*	205	190	180
Baseline vs. 9 months			
PTSC and ITT	0.03	0.12*	0.05
	[− 0.06 to 0.11]	[0.02 to 0.21]	[− 0.03 to 0.13]
*N*	201	189	178
Baseline vs. 15 months			
PTSC and ITT	− 0.02	0.03	− 0.002
	[− 0.12 to 0.09]	[− 0.09 to 0.15]	[− 0.11 to 0.11]
*N*	118	109	100

The usual care group is the reference category. We controlled for age, marital status, educational attainment, income, health insurance coverage, smoking and drinking behavior, health literacy, and numeracy. We have used multiple imputation methods to fill in missing observations for all covariates

*p < 0.05

**Table 4:** Fixed effects models for the 3 months, 9 months, and 15 months of balanced samples

Intention-to-treat analysis	5 servings of veg. s1q8ap	4 servings of fruit s1q8n	Watch Portion Sizes1q8q	Read food label s1q8t
	*b*/SE	*b*/SE	*b*/SE	*b*/SE
Baseline vs. 3 months				
PTSC and ITT	− 0.01	− 0.07	0.11*	0.09
	[− 0.11 to 0.09]	[− 0.17 to 0.03]	[0.01 to 0.21]	[− 0.01 to 0.18]
*N*	202	199	199	200
Baseline vs. 9 months				
PTSC and ITT	− 0.01	− 0.1	0.06	0.11*
	[− 0.11 to 0.09]	[− 0.20 to 0.01]	[− 0.04 to 0.16]	[0.02 to 0.20]
*N*	201	198	196	196
Baseline vs. 15 months				
PTSC and ITT	0.01	− 0.03	0.09	0.07
	[− 0.12 to 0.15]	[− 0.17 to 0.11]	[− 0.05 to 0.23]	[− 0.07 to 0.20]
*N*	116	113	114	115

The usual care group is the reference category. We controlled for age, marital status, educational attainment, income, health insurance coverage, smoking and drinking behavior, health literacy, and numeracy. We have used multiple imputation methods to fill in missing observations for all covariates

*p < 0.05

Stress levels declined for PTSC participants relative to the usual care group at 3 and 9 months from baseline, however, these results are not statistically significant. There is evidence that PTSC participants adopted healthy stress management techniques. Compared to the usual care group, PTSC participants adopted more adaptive techniques to reduce stress by 0.61 (CI 0.07, 1.14) at 3 months and by 0.68 (CI 0.13, 1.23) at 9 months, but this statistically significant difference disappeared by 15 months. PTSC participants did not change their use of non-adaptive techniques relative to the usual care group (See Table 5).

**Table 5:** Fixed effects models for the 3 months, 9 months, and 15 months of balanced samples

Intention-to-treat analysis	Stress level	Adaptive techniques	Non-adaptive techniques
	*b*/SE	*b*/SE	*b*/SE
Baseline vs. 3 months			
PTSC and ITT	− 0.14	0.61*	0.35*
	[− 0.52 to 0.24]	[0.07 to 1.14]	[0.01 to 0.69]
*N*	203	205	201
Baseline vs. 9 months			
PTSC and ITT	− 0.13	0.68*	0.26
	[− 0.51 to 0.25]	[0.13 to 1.23]	[− 0.08 to 0.59]
*N*	198	202	198
Baseline vs. 15 months			
PTSC and ITT	0.06	0.54	0.13
	[− 0.46 to 0.58]	[− 0.25 to 1.33]	[− 0.33 to 0.58]
*N*	117	118	115

The usual care group is the reference category. We controlled for age, marital status, educational attainment, income, health insurance coverage, smoking and drinking behavior, health literacy, and numeracy. We have used multiple imputation methods to fill in missing observations for all covariates

*p < 0.05

## Discussion

The results from the PTSC-RCT study indicate that the intervention contributed to a modest reduction in BP. However, it had no impact on BMI. The modest reduction in BP could be attributed to PTSC limited impact on health behaviors. The relationship between participation in the PTSC intervention and changes in participants’ BP and BMI in our study were not as strong as the relationships estimated in the prior pre and post evaluations of the PTSC program, or other community-based lifestyle change interventions targeting African American women [16, 24]. We note the possibility that differences in the outcomes observed in the community-based implementation of the program, versus the randomized controlled trial, may be partially attributable to the socioeconomic profile of those in the PTSC-RCT sample compared to their counterparts in the previous evaluations. The PTSC-RCT sample had significantly lower income and educational attainment compared to the women who have historically participated in the PTSC program.

The results observed among those who participated in the intervention group, as well as the size of the ITT group, illuminates the realities of intervention delivery and receipt among a largely low-income population. In our protocol paper, we noted the results of a brief survey administered to those in the ITT group, which sought to uncover some of the barriers to participation. Transportation, scheduling, and not feeling well were the most frequently cited responses. These experiences underscore two critical factors. First, that structural factors shaping the social environments and contexts of PTSC-RCT participants fundamentally affected the ability to participate in the program (the ITT group) and may have blunted the impact of the intervention (the PTSC intervention group). Although the women overwhelmingly appreciated the PTSC intervention, their ability to implement the lifestyle changes encouraged by PTSC may have been compromised by the realities of residing in structurally disadvantaged neighborhoods within the most impoverished Wards in Washington, D.C. A paucity of healthy food options [25] safe places to exercise, including food deserts, the lack of recreational facilities, poor housing conditions, inadequate and sometimes unsafe transportation, and other community-level factors may have undermined participants’ capacity to adopt strategies to prioritize their health [26]. Our study results appear to affirm the well-documented, profound connection between social determinants of health and hypertension management [6, 27, 28].

The second factor is related to the intersection of adverse social determinants of health that our study participants faced indicates the need to incorporate socially contextualized intervention strategies to meet the psychosocial, material, and health needs of a low-income population. PTSC participants faced constant threats to their ability to maintain their health. The constant pressures associated with residing in marginalized, disinvested communities influenced their self-reported stress, even as they attempted to adopt adaptive stress management techniques. Inasmuch as there were barriers to intervention receipt, there may have also been conceptual deficiencies in our deployment of the intervention for low-income women. The core components of the PTSC intervention (didactic training, peer support, and role modeling) were likely inadequate in promoting self-management of high blood pressure in the face of the previously noted adverse social determinants of health. Our results show that interventions intended to support low-income African American women must account for the preponderance of social risk factors they navigate. Enhancing the standard PTSC intervention with direct links to community-based resources, such as transportation, food, and other forms of assistance, may mitigate the impact of social risk factors on participants’ lives. In addition, it is possible that the certain elements of the program are more salient for low-income African American women than others—for instance, maintaining regular contact with others within the Sister Circles may offer invaluable social support and peer learning, with implications for sustaining newly acquired health behaviors.

### Strengths and limitations

A few aspects of this study need more explanation. First, the size of the ITT group hints at individual-level, intervention delivery, and implementation fidelity factors that affect our ability to conclusively determine the effectiveness of the PTSC intervention among study participants. Second, over half of the women in the PTSC-RCT sample had BP in the normotensive range at baseline. We did not exclude these women from the study at the request of the partnering FQHC, because they did not want to give women deemed eligible for participation an incentive to not take their BP medication on recruitment days so they could participate in our study. The inclusion of these women in the study may have reduced our ability to discern the true impact of the PTSC intervention on improved BP for women with uncontrolled hypertension. To address this concern, we estimated the model for women who were hypertensive at baseline. The results were similar, but we lost statistical power. Third, we did not use food diaries to measure changes in dietary habits or employ validated measures for physical activity and stress management techniques. Finally, the study enrollment of 339 was 70% of our target of 480 participants. This reduced our study’s expected statistical power and may be the primary source of our insignificant effect sizes. Our target enrollment was probably too ambitious given we were drawing from a relatively small population of 2216 potential participants which required enrolling one of every five eligible patients.

## Conclusions

Culturally tailored, community-based interventions can help low-income African American women with hypertension better manage their blood pressure. *Prime Time Sister Circles®* (*PTSC*) *program* is an example of how public health interventions design for the general population can be modified and packaged to effective promote lifestyle behavior changes among low-income populations with high needs. The *Prime Time Sister Circles®* (*PTSC*) *program* holds promise in motivating women to consider lifestyle changes that can help them prioritize their health. However, the *PTSC program*, and others like it, should consider booster interventions after a year to sustain the effects. Also, participants may need supplemental interventions that address health-related social risk factors, which may hinder participants’ ability adopt and sustain the lifestyle modifications needed to control blood pressure and improve their overall health.

### Supplementary Information

Below is the link to the electronic supplementary material.

Supplementary file1 (DOCX 74 kb)

## Data Availability

Data from the PTSC-RCT project will become available to researchers in the Spring 2024.

## Abbreviations

PTSC: Prime Time Sister Circles®
SBP: Systolic blood pressure
DBP: Diastolic blood pressure
ITT: Intent-to-treat
PSS: Perceived stress scale
