# Supplementary material for: The impact of the Prime Time Sister Circles® (PTSC) on blood pressure of low-income mid-life African American women in the United States
Source: J Public Health Policy. 2023 Oct 29;44(4):616–33. doi: 10.1057/s41271-023-00450-5 (PMC10709469; doi:10.1057/s41271-023-00450-5)
Supplement: Supplementary file 1 — Supplementary file1 (DOCX 74 kb) [file 41271_2023_450_MOESM1_ESM.docx]

**The Impact of the Prime Time Sister Circles® (PTSC) on Blood Pressure of Low Income Mid Life African American Women in the United States**

Darrell J. Gaskin, Hossein Zare, Chidinma A. Ibe, Manshu Yang, Wehmah Jones, Marilyn Gaston, Gayle Porter, Denise L. Woods, Michele Balamani, Nicole Jones, Vivienne A. Rose, Richard Allen Williams, Charles Rohde

**Supplementary Material**

**Table S1.** Impact of PTSC participation on change in systolic and diastolic blood pressure at 3 months, 9 months, and 15 months for hypertensive mid-life African American women who use an FQHC for their primary care.

| **Per Protocol Analysis** | SBP**^†^** | DBP**^‡^** | BMI§ |
| --- | --- | --- | --- |
|  | b/se | b/se | b/se |
| Baseline vs. 3-months | | | |
| PTSC and ITT | -2.96 | -2.19 | -0.06 |
|  | [-6.84-0.91] | [-4.75-0.37] | [-1.79-1.67] |
| N | **193** | **193** | **193** |
| Baseline vs. 9-months | | | |
| PTSC and ITT | -2.61 | -1.28 | 0.20 |
|  | [-6.39-1.17] | [-3.69-1.13] | [-1.51-1.91] |
| N | **195** | **195** | **195** |
| Baseline vs. 15-months | | | |
| PTSC and ITT | -3.01 | -3.68* | 0.37 |
|  | [-6.90-0.89] | [-6.58--0.79] | [-1.44-2.18] |
| N | **168** | **168** | **168** |

* p<0.05; ** p<0.01; *** p<0.001

The usual care group is the reference category. We controlled for age, marital status, educational attainment, income,

health insurance coverage, smoking and drinking behavior, health literacy, and numeracy.

We have used multiple imputation methods to fill in missing observations for all covariates.

**^†^**Systolic Blood Pressure (SBP)

**^‡^**Diastolic Blood Pressure (DBP)

§ Body Mass Index (BMI)

**Table S2.** Impact of PTSC participation on change in physical activity at 3 months, 9 months, and 15 months for hypertensive mid-life African-American women who use an

FQHC for their primary care

| **Per Protocol Analysis** | Exercise | Strength  Training | Pedometer  Use |
| --- | --- | --- | --- |
|  | b/se | b/se | b/se |
| Baseline vs. 3-months | | | |
| PTSC and ITT | -0.04 | 0.13* | 0.15** |
|  | [-0.13-0.06] | [0.03-0.24] | [0.06-0.25] |
| N | **174** | **161** | **151** |
| Baseline vs. 9-months | | | |
| PTSC and ITT | 0.02 | 0.15** | 0.07 |
|  | [-0.07-0.12] | [0.05-0.25] | [-0.02-0.15] |
| N | **171** | **161** | **151** |
| Baseline vs. 15-months | | | |
| PTSC and ITT | 0.01 | 0.10 | 0.01 |
|  | [-0.10-0.12] | [-0.03-0.24] | [-0.11-0.13] |
| N | **105** | **97** | **90** |

* p<0.05; ** p<0.01; *** p<0.001

The usual care group is the reference category. We controlled for age, marital status, educational attainment, income, health insurance coverage,

smoking and drinking behavior, health literacy, and numeracy.

We have used multiple imputation methods to fill in missing observations for all covariates.

**Table S3.** Fixed effects models for the 3 months, 9 months, and 15 months balanced samples.

| **Per Protocol Analysis** | 5 servings of Veg.  s1q8ap | 4 servings of fruit  s1q8n | Watch Portion Size  s1q8q | Read food label  s1q8t |
| --- | --- | --- | --- | --- |
|  | b/se | b/se | b/se | b/se |
| Baseline vs. 3-months |  |  |  |  |
| PTSC and ITT | -0.01 | -0.05 | 0.12* | 0.12* |
|  | [-0.12-0.10] | [-0.16-0.05] | [0.02-0.23] | [0.02-0.22] |
| N | **171** | **168** | **169** | **169** |
| Baseline vs. 9-months |  |  |  |  |
| PTSC and ITT | -0.01 | -0.07 | 0.08 | 0.14** |
|  | [-0.12-0.10] | [-0.18-0.04] | [-0.03-0.18] | [0.04-0.24] |
| N | **171** | **168** | **168** | **166** |
| Baseline vs. 15-months |  |  |  |  |
| PTSC and ITT | -0.01 | -0.003 | 0.12 | 0.11 |
|  | [-0.16-0.14] | [-0.15-0.15] | [-0.03-0.26] | [-0.03-0.25] |
| N | **103** | **101** | **102** | **102** |

* p<0.05; ** p<0.01; *** p<0.001

The usual care group is the reference category. We controlled for age, marital status, educational attainment, income, health insurance coverage,

smoking and drinking behavior, health literacy, and numeracy.

We have used multiple imputation methods to fill in missing observations for all covariates

**Table S4.** Fixed effects models for the 3 months, 9 months, and 15 months balanced samples.

| **Per Protocol Analysis** | Stress level | Adaptive  Techniques | Non-Adaptive Techniques |
| --- | --- | --- | --- |
|  | b/se | b/se | b/se |
| Baseline vs. 3-months | | | |
| PTSC and ITT | -0.21 | 0.65* | 0.28 |
|  | [-0.61-0.19] | [0.07-1.22] | [-0.08-0.65] |
| N | **172** | **174** | **170** |
| Baseline vs. 9-months | | | |
| PTSC and ITT | -0.11 | 0.74* | 0.26 |
|  | [-0.52-0.30] | [0.15-1.33] | [-0.11-0.62] |
| N | **169** | **172** | **168** |
| Baseline vs. 15-months | | | |
| PTSC and ITT | -0.15 | 0.63 | 0.09 |
|  | [-0.72-0.42] | [-0.23-1.49] | [-0.39-0.57] |
| N | **104** | **105** | **102** |

* p<0.05; ** p<0.01; *** p<0.001

The usual care group is the reference category. We controlled for age, marital status, educational attainment, income, health insurance coverage,

smoking and drinking behavior, health literacy, and numeracy.

We have used multiple imputation methods to fill in missing observations for all covariates

Assessed for eligibility (n= 359)

Excluded (n= 18)

♦  Declined to participate (n= 18)

♦  Other reasons (n=0)

Randomized (n=341)

## Enrollment

**Figure S1.** PTSC Study CONSORT Flow Diagram

Lost to follow-up (n=21)

Discontinued usual care (n=0)

Lost to follow-up (n=4)

Discontinued usual care (n=0)

Analysed (n=100)

♦ Excluded from analysis (n=0)

Lost to follow-up (n=25)

Discontinued usual care (n=0)

Analysed (n=140)
♦ Excluded from analysis (n=0)

Analysed (n=138)
♦ Excluded from analysis (n=0)

## Analysed 15 Months

Analysed (n=142)
♦ Excluded from analysis (n=0)

Analysed (n=75)

♦ Excluded from analysis (n=0)

Lost to follow-up (n=22)

Discontinued usual care (n=0)

## 15 Month Follow-Up

## Analysed 9 Months

Analysed (n=96)

♦ Excluded from analysis (n=0)

Intervention group lost to follow-up (n=2)

Intent-to-treat group lost to follow-up (n=0)

## 9 Month Follow-Up

## Analysed 3 Months

## 3 Month Follow-Up

Intervention group lost to follow-up (n=12)

Intent-to-treat group lost to follow-up (n=64)

Allocated to PTSC intervention (n=216)

♦ Received allocated intervention (n=103)

♦ Did not receive allocated intervention (“intent-to-treat”) (n=113)

Allocated to usual care (n=125)

♦ Received usual care (n=125)

♦ Did not receive usual care (n=0)

## Allocation
